# Identification of a novel TLR7 gain-of-function variant that underlies systemic lupus erythematosus

**DOI:** 10.70962/jhi.20250194

**Published:** 2026-02-24

**Authors:** Aiswarya Sethumadhavan, Charles Mariasoosai, Natsuko Yamakawa, Nicolas Chamberlain, Matthew A. Deardorff, Shintaro Funaki, Takaki Asano, Jean-Laurent Casanova, Hedieh Torabifard, Bertrand Boisson, Eric Meffre

**Affiliations:** 1 Department of Immunology and Rheumatology, Stanford University School of Medicine, Stanford, CA, USA; 2 Department of Chemistry and Biochemistry, The University of Texas at Dallas, Richardson, TX, USA; 3 Department of Immunobiology, Yale University School of Medicine, New Haven, CT, USA; 4 Departments of Pathology and Pediatrics, Children’s Hospital Los Angeles Keck School of Medicine, University of Southern California, Los Angeles, CA, USA; 5 Department of Pediatrics, Hiroshima University Graduate School of Medicine and Health Sciences, Hiroshima, Japan; 6 Department of Genetics and Cell Biology, Research Institute for Radiation Biology and Medicine, Hiroshima University, Hiroshima, Japan; 7 St. Giles Laboratory of Human Genetics of Infectious Diseases, Rockefeller Branch, The Rockefeller University, New York, NY, USA; 8 Laboratory of Human Genetics of Infectious Diseases, Necker Branch, INSERM U1163, Necker Hospital for Sick Children, Paris, France; 9 Imagine Institute, University of Paris, Paris, France; 10 Howard Hughes Medical Institute, New York, NY, USA

## Abstract

Gain-of-function (GOF) variants in human TLR7 have recently been reported in 11 cases, six of which were diagnosed with systemic lupus erythematosus (SLE). We have identified the X-linked L840R *TLR7* variant in hemizygosity in a male patient with SLE and in heterozygosity in his clinically asymptomatic mother. The leucine 840 is located at the first amino acid of TLR7 transmembrane domain and is conserved across various species. The L840R substitution is predicted to be deleterious by various scoring algorithms and may therefore affect TLR7 function. Molecular dynamics simulations of TLR7–UNC93B1 interactions revealed that R840 alters nearby amino acids interactions, resulting in increased hydrogen bond between E834 of TLR7 with R157 of UNC93B1. Finally, the L840R TLR7 variant has increased activity compared with WT, as measured with a nuclear factor κB (NF-κB)–specific luciferase reporter upon stimulation with TLR7 agonist R848. Hence, hemizygosity for L840R confers GOF for NF-κB activation and underlies SLE by potentially increasing TLR7 binding to UNC93B1.

## Teaser

Sethumadhavan, Mariasoosai, Yamakawa et al. report a novel TLR7 gain-of-function (GOF) variant associated with autoimmunity in a male patient, which further highlights TLR7's essential role in SLE pathogenesis. The L840R mutation may increase TLR7 binding with UNC93B1, which is a molecular mechanism that was not previously described for other TLR7 GOF or UNC93B1 mutations.

## Introduction

Mouse models have shown that Toll-like receptors (TLRs) that bind nucleic acids play an essential role in the development of autoimmunity (1). TLR7, which binds single-stranded RNA, triggers autoimmunity by promoting autoreactive B cell activation and the secretion of autoantibodies (2, 3). As a consequence, TLR7 deficiency results in a marked decrease in autoimmune manifestations in multiple autoimmune mouse models (1). Conversely, mice carrying the Y-linked autoimmune accelerating locus, which have an additional copy of the *TLR7* gene on the Y chromosome that resulted from a segment translocated from the X chromosome, develop fatal lupus-like disease (4, 5). This demonstrates that enhanced TLR7 function promotes autoimmunity and lupus-like disorders in mice.

In humans, polymorphisms in the *TLR7* gene and the analysis of its expression suggest an involvement of this receptor in the pathogenesis of several autoimmune diseases, including systemic lupus erythematosus (SLE) and Sjogren’s syndrome (6, 7, 8). The analysis of human inborn errors associated with the early development of autoimmunity and enhanced type I interferon production further revealed the importance of the TLR7 pathway for SLE in humans (9, 10, 11). Indeed, TLR7 gain-of-function (GOF) mutations were recently reported in patients with early onset of SLE, which demonstrates the key role of enhanced TLR7 function in this disease (12, 13, 14, 15). In addition, TLR7 GOF mutations also induced other autoimmune disorders, including immune thrombocytopenic purpura (ITP), autoimmune hemolytic anemia, and neurological diseases (12, 13, 14, 15, 16). The Y264H TLR7 GOF missense mutation is in the TLR7 ligand-binding domain and was shown to enhance guanosine and 2′,3′, cGMP TLR7 ligand affinity for their receptor (12). The L267P TLR7 GOF mutation is only three amino acids apart from the previous mutation and may also impact TLR7 binding to its ligands (13). In contrast, the F506S, F507L, F507S, and L528I TLR7 GOF mutations are in the TLR7 homo-dimerization domain and enhance TLR7 signaling by a yet unappreciated mechanism, whereas the G818V mutation appears to activate TLR7 independently of ligand binding (14, 15, 16). We searched for additional patients with other TLR7 GOF variants, which may reveal novel molecular mechanisms driving autoimmunity in humans.

## Results

### Clinical and laboratory data

**Figure 1.**
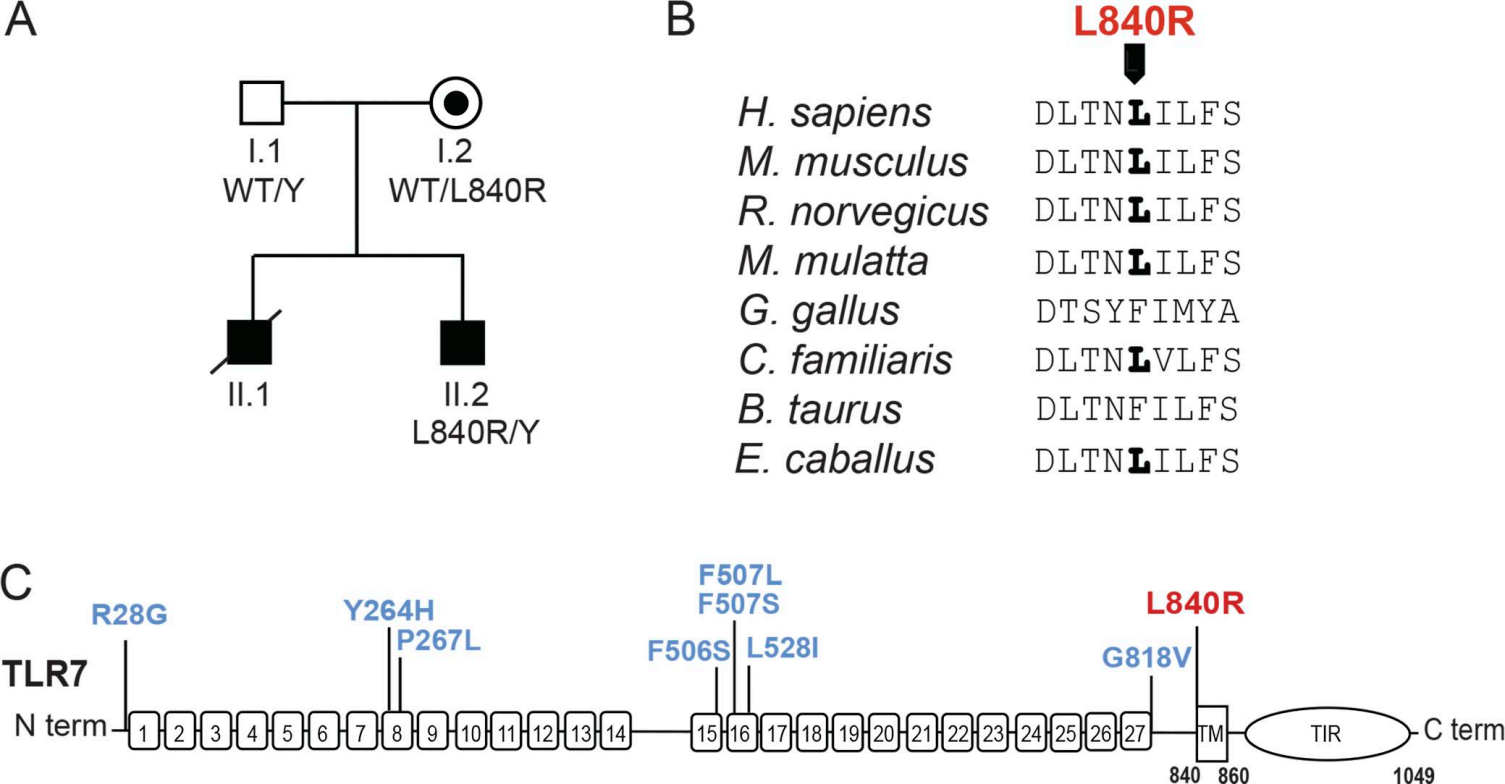
**TLR7 GOF mutations in patients with SLE. (A)** Family pedigree. Circles and squares indicate female and male family members, respectively. Filled symbols indicate patients with SLE. TLR7 genotypes are provided under each symbol, when available. The line across II:1 indicates deceased status. **(B)** Alignment of TLR7 primary sequences of indicated species; bold letter indicates conservation of residue. **(C)** Representation of the structure of TLR7; numbers indicate endosomal LRR regions; TM indicates the transmembrane domain, while the toll/interleukin-1 receptor (TIR) signaling domain is cytoplasmic. Previously reported TLR7 GOF mutations are indicated in blue on the top, while the novel mutation reported in this study is in red.

Patient II.2 is a 22-year-old male born to non-consanguineous parents of Asian ancestry (Fig. 1). He first presented at 3 years old with mucocutaneous bleeding, a platelet count <10 k/μl, and a microcytic reticulocytopenic anemia. He was diagnosed with ITP and iron deficiency anemia and treated with intravenous immunoglobulins and Rh immune globulin (WinRho). At age 13, he had a persistent cervical lymphadenopathy, a mild neutropenia (1,200/μl), a normocytic anemia (11.9 g/dl), and a platelet count of 82 k/μl. The lymphadenopathy, anemia, and neutropenia resolved and were felt to be virally induced. A moderate thrombocytopenia persisted, and the presence of antiplatelet antibodies supported an immune etiology diagnosed as chronic ITP that led to SLE. Supporting criteria for the diagnosis of SLE included autoimmune thrombocytopenia, proteinuria, positive dilute Russell viper venom test, antinuclear antibody (ANA) positive (1:320), and double-stranded DNA (dsDNA) binding-positive (54 IU/ml, normal 0–24.9 IU/ml). C3 complement levels were low (89 mg/dl, normal 90–187 mg/dl), but he displayed normal C4 concentrations (21.0 mg/dl, normal 16–45 mg/dl), whereas his CH50 was low (70 U/ml, normal 104–356 U/ml). Anti-smith (SM), anti-RNP, anti-Sjögren’s Syndrome-related antigen A (SS-A), and anti-Sjögren’s Syndrome-related antigen B (SS-B) antibodies were absent, but antiplatelet IgG was strongly positive, and his IgE level was elevated (377 IU/ml, normal 0–200 IU/ml). He is also positive for antithyroid peroxidase antibodies with an elevated thyroid-stimulating hormone (TSH) but normal free T4. He had normal growth and development with no abnormalities on physical exam. He was treated with hydroxychloroquine, which resolved his ITP, proteinuria, and serologic disease. A brother (II.1) had a hemolytic anemia starting at 1 year of age and was diagnosed with SLE when he was 19 years old. He died at age 29 from complications of a stroke secondary to SLE before genotyping of family members was performed.

### Molecular data

Exome sequencing at 14 years age identified a hemizygous *TLR7* c.2519T > G (p. Leu840Arg) variant in patient II.2 that was inherited from his mother who was clinically asymptomatic (Table 1 and Fig. 1 A). We could not assess the presence of this TLR7 genotype in patient II.1 because he was already deceased. The leucine at residue 840 is conserved in TLR7 across various species and is located at the first amino acid of the transmembrane domain of TLR7 (Fig. 1, B and C). The L840R substitution was not present on gnomAD v4 database as no other variation at this position (Table 1). This amino acid replacement may affect TLR7 function, as the L840R substitution is predicted to be deleterious by combined annotation dependent depletion (CADD) (22.5) and sorting intolerant from tolerant (SIFT) (0.01) with a mutation significance cutoff of 16.3, which suggests that this TLR7 variant could be causal of SLE (Table 1).

**Table 1.** Clinical characteristics of two male patients with SLE with one confirmed for carrying a rare *TLR7* variant

Patients	TLR7 mutation	Allele frequency (gnomAD)	Mutation effect (SIFT)	Mutation effect CADD (MSC)	Variant effect predictor score	Current age	Age at onset	Autoimmune manifestation	Other
II.1	​	​	​	​	​	29 years (deceased)	1 year	SLE and autoimmune hemolytic anemia	Cerebrovascular accident
II.2	c.2519T > G p.L840R	Not present	Deleterious (0.01)	Deleterious 22.5 (16.3)	Likely pathogenic 0.599	22 years	3 years	SLE, ITP, and cervical lymphadenopathy	Iron deficiency anemia

MSC, mutation significance cutoff.

**Figure 2.**
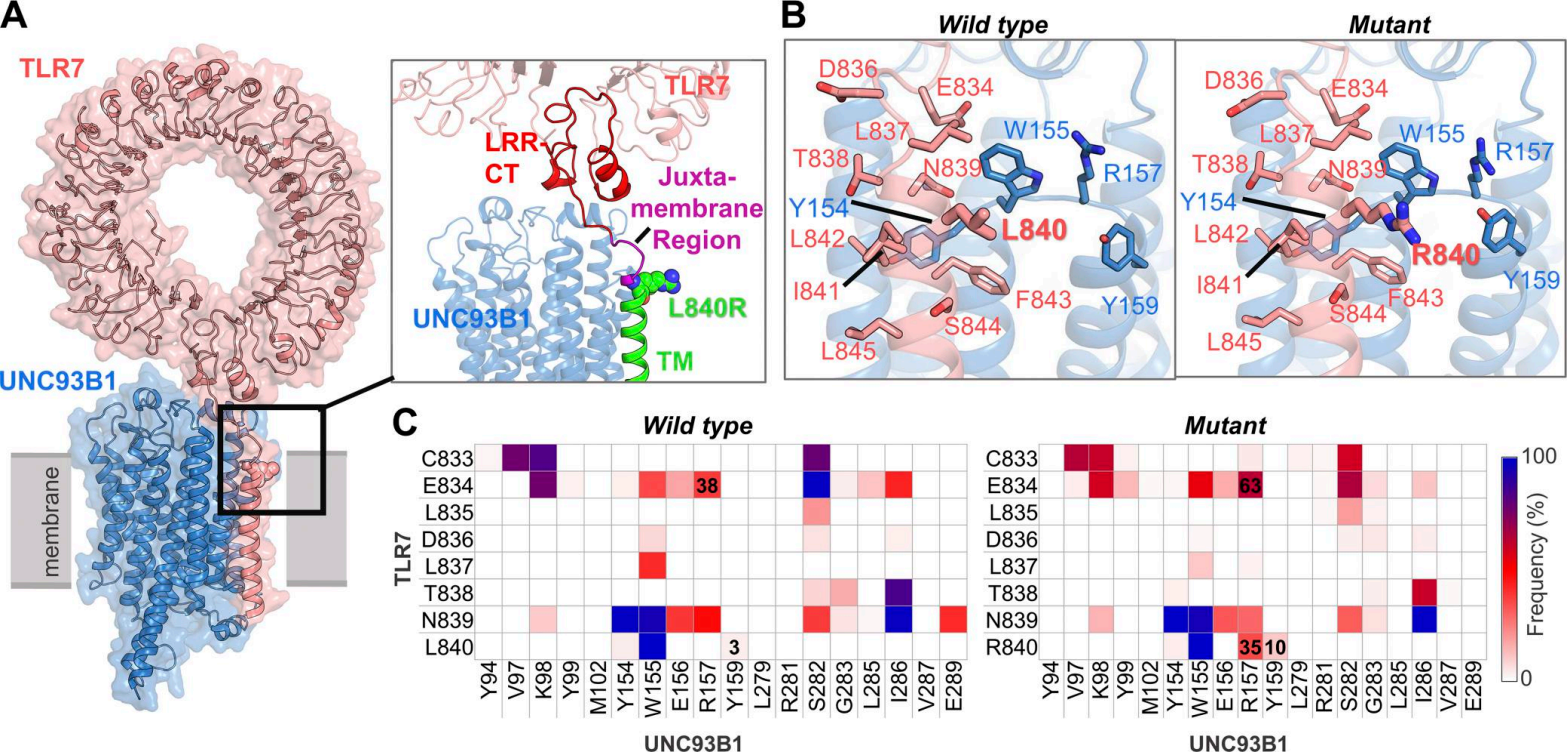
**L840R substitution rearranges TLR7 interface interactions with UNC93B1. (A)** Complex structure of TLR7 with UNC93B1. TLR7 is shown in coral, and UNC93B1 is depicted in blue. The large ring-shaped LRR domain is positioned on the luminal side of the membrane. TLR7 interacts with UNC93B1 through the leucine-rich repeat C-terminal (LRR-CT) motif, the luminal juxtamembrane region, and the transmembrane (TM) helix. **(B)** Representation of the residue 840 in both wild-type and mutant TLR7–UNC93B1 complex. **(C)** Heatmaps showing the contact frequency between the juxtamembrane region of TLR7 and the transmembrane helices of UNC93B1, calculated from MD simulations. Values are given in the plot to highlight the most significant differences that are boxed. In the mutant, R840 of TLR7 forms an additional contact with R157 of UNC93B1. The overall nonpolar contacts at the interface are moderately increased for the mutant at the L840R mutation site.

**Figure S1.**
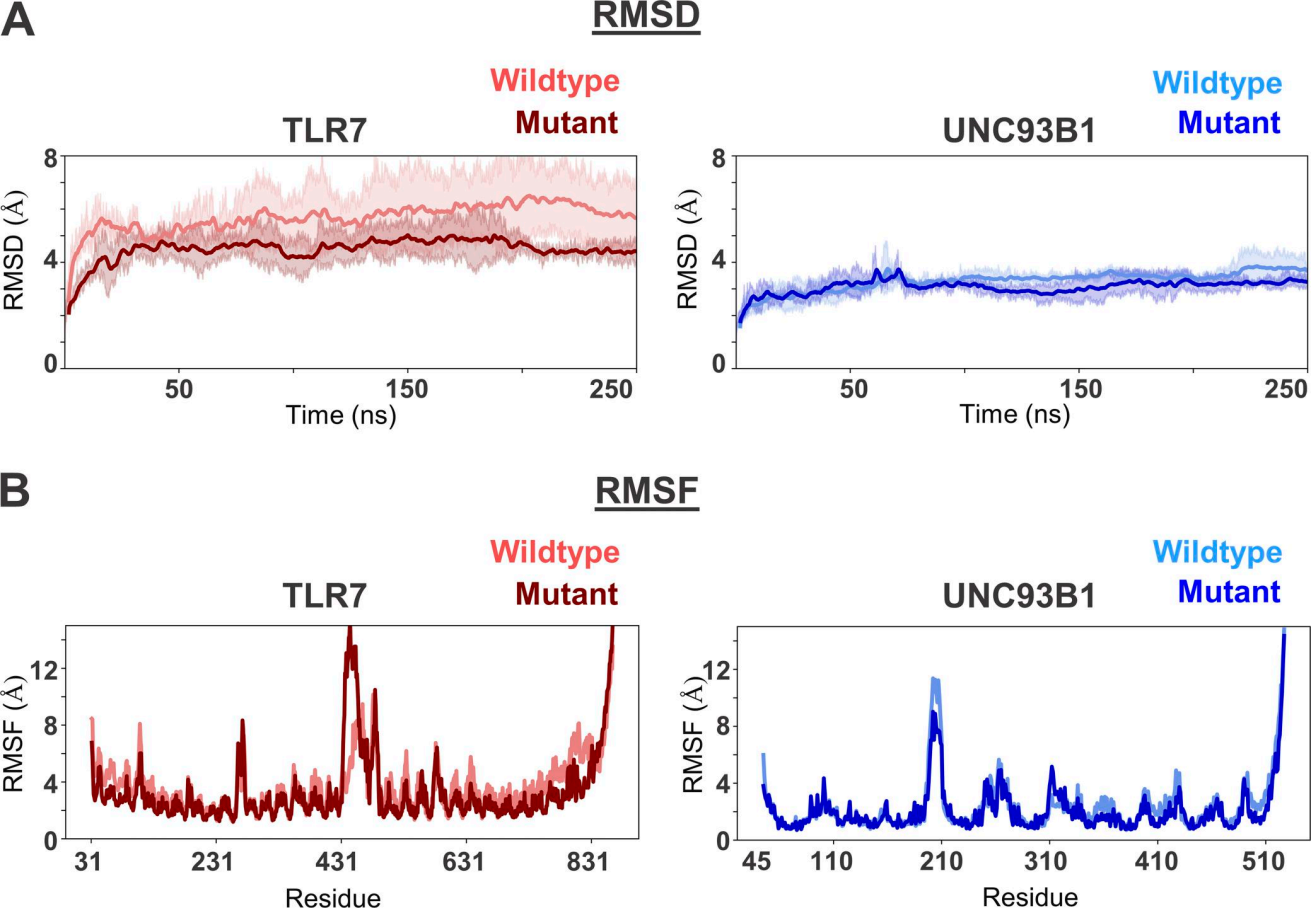
**RMSD and RMSF plots of TLR7 and UNC93B1 within the complex. (A)** The RMSD plot of the protein backbone reflects structural stability over the course of the simulation. The lower RMSD observed for the mutant TLR7 indicates that its structural fold is more stable compared to the wild type. **(B)** RMSF plots show the residue-level flexibility of TLR7 and UNC93B1, with both proteins exhibiting similar fluctuation profiles across their respective residues.

We explored this hypothesis by analyzing the impact of the L840R substitution on the complex structure of TLR7 interacting with UNC93B1 by performing molecular dynamics (MD) simulations of the L840 (wild type) versus R840 (mutant) TLR7–UNC93B1 complexes. The L840R substitution is located at the junction between the juxtamembrane region and the transmembrane helix of TLR7 (Fig. 2 A). The cryo-EM structure of the TLR7–UNC93B1 (PDB ID: 7CYN) complex was used as the initial model, and the L840R substitution was introduced to generate the mutant complex for MD simulations (Fig. 2 B). Both the wild-type and mutant TLR7 formed stable complexes with UNC93B1 throughout the simulation period, with comparable binding energies of −199.85 ± 47.23 kcal/mol and −197.62 ± 62.45 kcal/mol, respectively. The root mean square deviation (RMSD) of the mutant TLR7–UNC93B1 complex was slightly lower than that of the wild type, suggesting that the mutant TLR7 and UNC93B1 form a more stable complex (Fig. S1 A). In terms of residue flexibility, both the wild-type and mutant complexes exhibited comparable root mean square fluctuation (RMSF) profiles (Fig. S1 B).

**Figure S2.**
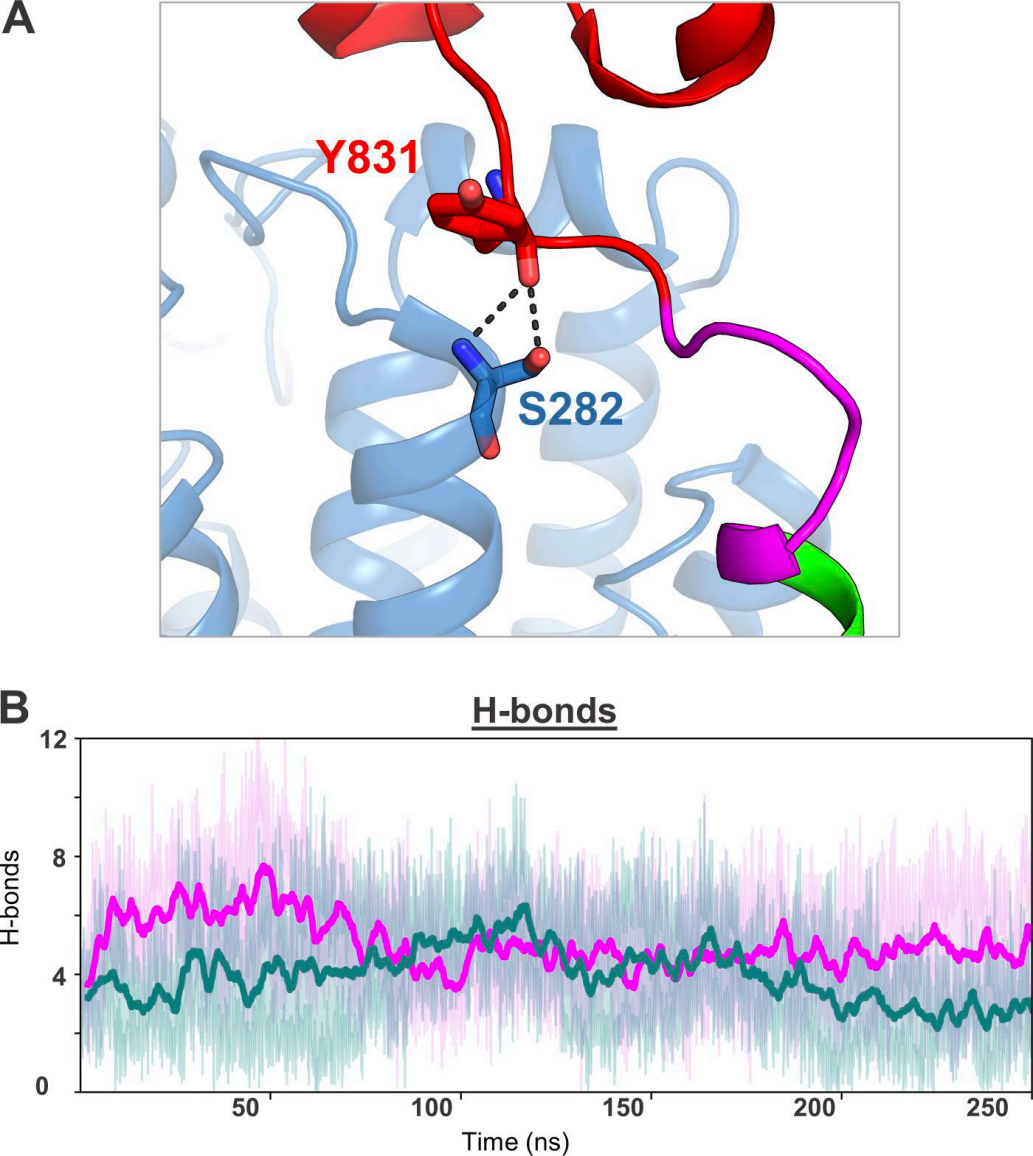
**Hydrogen bonds between TLR7 and UNC93B1. (A)** Representation of the hydrogen bond observed between TLR7 and UNC93B1 in the cryo-EM structure, specifically between Y831 of TLR7 and S282 of UNC93B1. In the wild-type TLR7–UNC93B1 complex, the backbone carbonyl oxygen of Y831 in TLR7 interacted with both the backbone nitrogen and the side chain hydroxyl group of S282 in UNC93B1, with occupancy rates of 66.47 and 7.71%, respectively, which changed to 36.66 and 10.35% in the mutant complex. **(B)** Average number of hydrogen bonds formed between NC93B1 and either the wild-type or mutant (L840R) TLR7 during the MD simulations.

While the N-terminal six-helix bundle of UNC93B1 interacts with TLR7 primarily through extensive hydrophobic contacts (17), we analyzed the polar and nonpolar interactions between wild-type and mutant TLR7 and UNC93B1. Both wild-type and the mutant complexes formed an average of five hydrogen bonds at their TLR7–UNC93B1 interface during the simulation (Fig. S2). The only hydrogen bond observed in the cryo-EM structure, between Y831 in TLR7 and S282 in UNC93B1, was consistently maintained in simulations for both complexes (Fig. S2 and Table S1). While the L840R substitution introduces a polar residue at position 840, we found that the R840 does not form any significant polar interactions in the TLR7–UNC93B1 complex.

**Figure 3.**
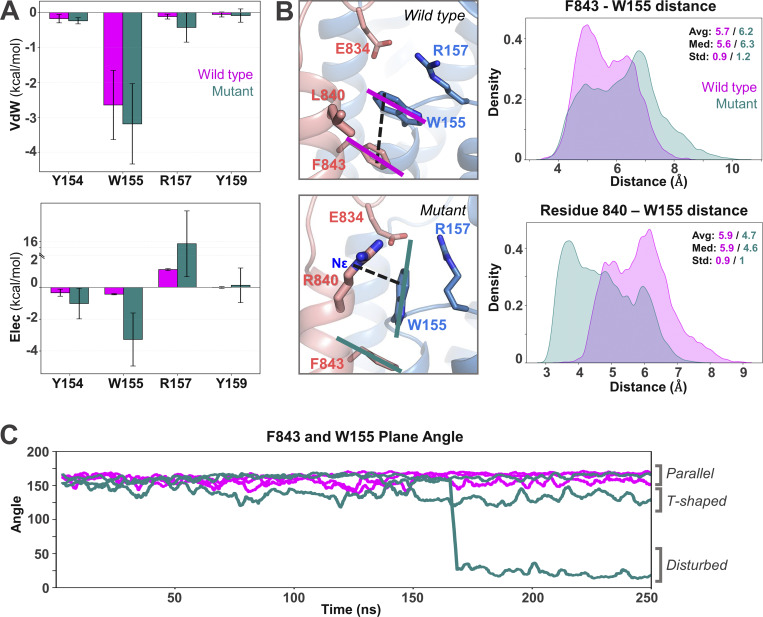
**R840 alters TLR7–UNC93B1 interactions. (A)** vdW and Elec interaction energies between wild-type L840 or mutant R840 and the interacting residues from UNC93B1. **(B and C)** (B) The π–π interaction between F843 of TLR7 and W155 of UNC93B1, and the π–cation interaction between R840 and W155, is shown on the left side panel. The black dashed lines indicate the interaction distance between W155, F843, and R840, and the solid lines (magenta and teal color) indicate the planes of the aromatic rings used to calculate the plane angle. Corresponding density plots of the interaction distances are on the right panel, and the orientation angles are plotted in the line plot (C). In the line plot, multiple lines of same color represent replicates. In the wild-type TLR7–UNC93B1 complex, L840 is positioned near F843 of TLR7 and W155 of UNC93B1. In MD simulations, L840 slightly shifts away from W155, favoring a stable π–π interaction between F843 and W155 in the wild type. In contrast, the R840 mutation forms a π–cation interaction with W155, disrupting the π–π interaction between F843 and W155, causing it to either be lost or shift into a T-shaped π–π interaction. Both ∼20° and ∼160° plane angles in the mutant indicate parallel rings; however, the rings slide and become displaced, increasing the distance between them and ultimately leading to loss of interaction.

**Figure S3.**
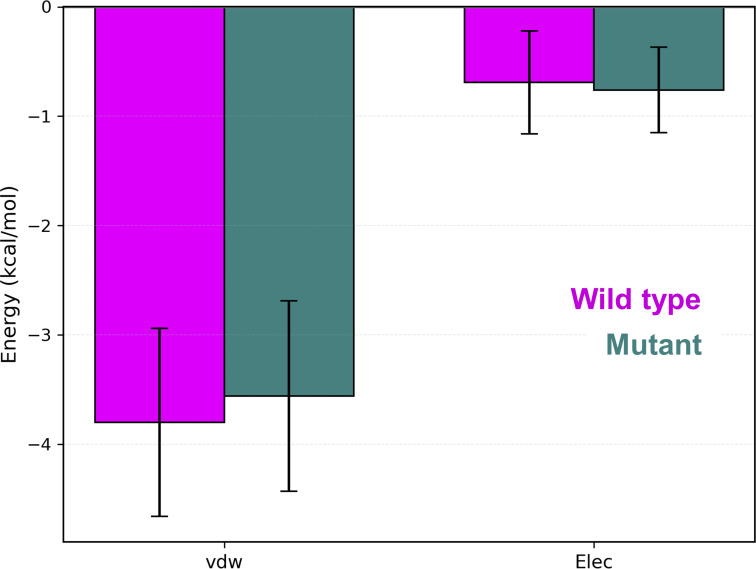
vdW and Elec interaction energies between F834 of TLR7 with W155 of UNC93B1.

To evaluate nonbonded interactions, we calculated residue–residue contact frequencies at the TLR7–UNC93B1 interface. The juxtamembrane region of mutant TLR7 showed more distributed contacts with UNC93B1 compared to the wild type (Fig. 2 C). Notably, the residue 840 (L840/R840) of TLR7 remained in constant contact with W155 of UNC93B1 in both the wild-type and mutant complexes. In the wild-type complex, L840 of TLR7 formed contacts with Y154, W155, and Y159 of UNC93B1, whereas in the mutant complex, R840 of TLR7 maintained contacts with Y154, W155, and Y159 and formed a new contact with R157 of UNC93B1 (Fig. 2 C). We also calculated the electrostatic (Elec) and van der Waals (vdW) interaction energies between L840/R840 and the UNC93B1 residues in contact. At the mutation site, both Elec and vdW energies were favorable for mutant R840 compared to L840 for all interacting residues, except for the unfavorable Elec interaction with R157 in the mutant complex (Fig. 3 A). L840 in the wild-type complex does not form any significant Elec interactions, but F843 of TLR7 forms a π–π interaction with W155 of UNC93B1 (Fig. 3, B and C). In contrast, R840 in the mutant complex forms a cation–π interaction with W155 (Fig. 3, B and C). The stability of the π–π and cation–π interactions was assessed by calculating the center of mass distance and angle between the aromatic rings of F843 and W155, as well as the center of mass of W155’s aromatic ring in UNC93B1 and the Nε atom of R840 in TLR7, respectively (Fig. 3, B and C). The normalized density plots of the distances and the line plots of the angles for the π–π interaction between F843 of TLR7 and W155 of UNC93B1 along with visualizing the MD trajectories indicate that this interaction formed a parallel π–π interaction in the wild-type complex, whereas it is either lost or transformed to a T-shaped π–π interaction in the mutant complex (Fig. 3, B and C). Although, these specific π–π interactions do not show any significant differences in the interaction energies (Fig. S3), the change of orientation in the mutant complex along with the repulsion from R840 pushes R157 of UNC93B1 toward the luminal side, resulting in a twofold increase in hydrogen bond frequency with E834 in the leucine-rich repeat (LRR)-CT domain of TLR7 (Fig. 2 C and Table S1). We conclude that the L840R substitution does not induce major conformational changes in the complex and leads to dispersed interface contacts between TLR7 and UNC93B1, whereas it induces a high interaction affinity between the two proteins at the site of mutation.

### The L840R missense mutation is a novel TLR7 GOF mutation associated with SLE

**Figure 4.**
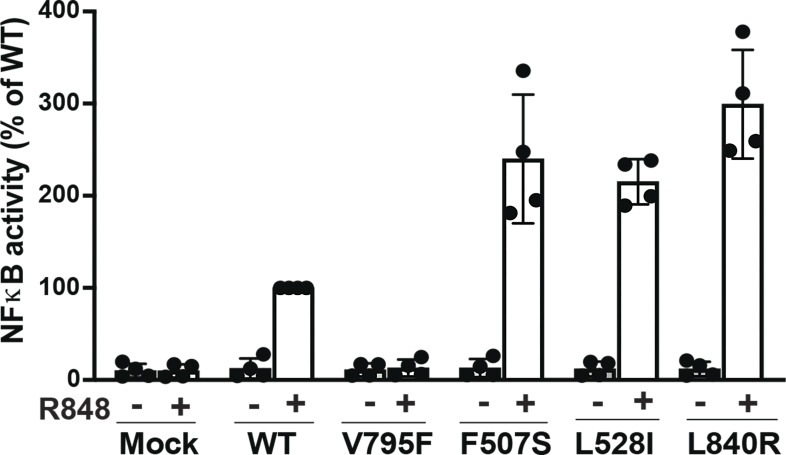
**Evaluation of the L840R TLR7 variant function.** Luciferase assay using HEK293T cells transfected with the pGL4.32 luciferase reporter construct and an expression vector for *Renilla* luciferase together with empty vector (Mock), or vectors expressing wild type (WT), LOF V795F TLR7 (negative control), GOF F507S and GOF L528I TLR7 (positive controls), or the L840R TLR7 variant stimulated or not by R848 TLR7 ligand. The results of four independent experiments are represented.

To evaluate the impact of the L840R substitution on TLR7 function, we tested the function of the L840R TLR7 after co-transfection with a nuclear factor κB (NF-κB)–specific luciferase reporter and measured luciferase activity upon stimulation with R848, an agonist of both TLR7 and TLR8 as previously described (Fig. 4) (18). Comparisons were made with wild-type TLR7, the loss-of-function (LOF) V795F TLR7 mutation as negative control (18), and two previously reported TLR7 GOF mutations (F507S and L528I) as positive controls (14). We found increased NF-κB induction by the two GOF TLR7 variants and the L840R TLR7 variant compared to wild type when cells were stimulated by R848, whereas the LOF V795F TLR7 variant failed to induce NF-κB as expected (Fig. 4). Thus, the L840R missense mutation is a novel TLR7 GOF mutation associated with SLE.

## Discussion

We have characterized a novel *TLR7* gene mutation in a male patient suffering from SLE in combination with either autoimmune hemolytic anemia or ITP. These autoimmune diseases developed early in life and were previously reported in other patients with TLR7 GOF mutations (12, 13, 14, 15). It is likely that the patient’s brother, who also suffered from early onset SLE and died before genotyping could be performed, was also hemizygous for this TLR7 mutation. We do not have any information about the mother carrier, suggesting that she is clinically asymptomatic despite being heterozygous carrier of the TLR7 GOF variant. The report of another asymptomatic mother with a heterozygous autosomal dominant G325C GOF variant in UNC93B1 that was also found in her daughter who developed early onset SLE suggests that other additional factors such as skewed X-chromosome inactivation or the presence of a protective allele may antagonize the effect of either *TLR7* or *UNC93B1* mutations (19). In the absence of a blood sample to assess X-chromosome inactivation and serum autoantibodies in the patient’s asymptomatic mother, it remains unclear whether the allele follows an X-linked dominant (with incomplete penetrance in female) or X-linked recessive mode of inheritance. The L840R TLR7 GOF mutation is not in the extracellular LRR domain as other previously reported TLR7 GOF mutations but is located at the beginning of TLR7 transmembrane domain, which suggests that it is not likely to affect either TLR7 ligand affinity or homo-dimerization (12, 13, 14, 15, 16).

Indeed, we found that the L840R substitution may promote autoimmunity by increasing TLR7 binding with UNC93B1, which is a chaperone that regulates TLR7 intracellular trafficking and signaling. Indeed, the arginine introduced at position 840 reduces the distance between TLR7 and W155 in UNC93B1 and increases the frequency of the hydrogen bond between E834 of TLR7 and R157 of UNC93B1. Strikingly, both W155 and R157 in loop 3 of UNC93B1 have previously been shown to play an important role in mediating TLR7 function, and our data now suggest that they are likely involved in TLR7/UNC93B1 interactions (17, 20). By analogy to the previously reported D34A UNC93B1 mutation in mice, increased L840R TLR7 binding to UNC93B1 may favor TLR7 export from the endoplasmic reticulum (ER) to the endosome and increase its signaling (21, 22). In addition, UNC93B1 also plays an important role in stabilizing and promoting TLR7 expression (23). The importance of TLR7/UNC93B1 axis in monogenic autoimmune diseases is highlighted by the description of several UNC93B1 mutations that enhanced TLR7 function (19, 20, 24, 25). However, none of these mutations enhanced binding to TLR7 but instead often decreased the mechanisms that terminate TLR7 signaling (19, 20, 24, 25). Of note, the E92G mutation in UNC93B1, which increases TLR7 responses, was shown to decrease UNC93B1 binding to TLR7 but also affects termination of TLR7 signaling by decreasing synthenin1 recruitment (24, 26). Hence, diverse alterations in UNC93B1 and TLR7 interaction may lead to enhanced TLR7 function.

In summary, we reported a novel *TLR7* GOF variant associated with autoimmunity in a male patient, which further highlights the essential role played by TLR7 in SLE pathogenesis. This new TLR7 GOF mutation may promote autoimmune disease via increased TLR7 binding with UNC93B1, which is a molecular mechanism that was not previously described for other *TLR7* GOF or *UNC93B1* mutations.

## Materials and methods

### Genetic studies

Exome sequencing was performed and analyzed using bioinformatics pipeline CES version 1.0 as previously described (27). Bioinformatics pipeline CES version 1.0 was developed incorporating NovoAlign (Novocraft) for read alignment, Picard (Broad Institute) for marking duplicates, and Genome Analysis Toolkit (Broad Institute) (27). Best practices for UnifiedGenotyper, with no parameter modifications, was used for variant calling (reference sequence: hg19 GRCh37) and variant filtering based on read depth (≥5×). Additional information is provided in Supplemental materials and methods at the end of the PDF.

### Structural analysis

The solved cryo-EM structure of TLR7 in complex with UNC93B1 was used as the input for modelization (PDB ID: 7CYN) (17). L840 of TLR7 is mutated to R to prepare the mutant structure. The protein in a mammalian ER membrane model system was constructed using CHARMM-GUI (28).

All simulations were run on a single Graphical processing unit using Amber20 Compute Unified Device Architecture version of particle-mesh Ewald MD (29). We used CHARMM36m parameter set for the protein and CHARMM36 force field for lipid molecules and TIP3P water models for water molecules (30, 31).

All analyses and binding energy calculations were performed using the cpptraj (32) and MMPBSA (33) programs from the AmberTools23, and Visual Molecular Dynamics package (VMD 1.9.3) (34), Matplotlib Python library, and PyMOL (2.3.2) packages were used for plots and molecule representation (35). Simulation input files and trajectories are uploaded to the Zenodo repository (https://doi.org/10.5281/zenodo.16541351). Additional detailed information about the structural analysis is provided in Supplemental materials and methods at the end of the PDF.

### Luciferase reporter assay

HEK293T cells, which have no endogenous TLR7 expression, were transfected as previously described (18). After 24 h, the transfected cells were stimulated or not with 50 ng/mL R848 (Resquimod) for activation via TLR7/8 (Invivogen) for 24 h. Relative luciferase activity was then determined by normalizing the values against the firefly:*Renilla* luciferase signal ratio.

### Statistical analysis

Statistical analysis was performed using GraphPad Prism software, version 9.4.1 (GraphPad Software). Statistical significance between groups was determined by Student’s *t* tests. A P value of <0.05 was considered significant.

### Online supplemental material

Supplementary information includes Supplementary materials and methods at the end of the PDF, three supplementary figures (Figs. S1, S2, and S3), and a supplementary table (Table S1). Fig. S1 shows RMSD and RMSF plots of TLR7 and UNC93B1 within the complex. Fig. S2 shows hydrogen bonds between TLR7 and UNC93B1. Fig. S3 shows vdW and Elec interaction energies between F834 of TLR7 with W155 of UNC93B1. Table S1 shows frequency of H-bonds formed at the interface of TLR7 and UNC93B1.

## Supplementary Material

Table S1 shows shows frequency of H-bonds formed at the interface of TLR7 and UNC93B1.

## Data Availability

The data underlying Figs. 1, 2, 3 and 4 and Table 1 are available in the published article and its online supplemental material. No datasets were generated or analyzed during the current study. The TLR7–UNC93B1 structures, input coordinates, and simulation parameter files are uploaded to the Zenodo repository (https://doi.org/10.5281/zenodo.16541351).
