# Supplementary material for: Identification of a novel TLR7 gain-of-function variant that underlies systemic lupus erythematosus
Source: J Hum Immun. 2026 Feb 24;2(3):e20250194. doi: 10.70962/jhi.20250194 (PMC12931374; doi:10.70962/jhi.20250194)
Supplement: Table S1 — shows shows frequency of H-bonds formed at the interface of TLR7 and UNC93B1. [file jhi_20250194_tables1.docx]

**Table S1.** Frequency of H-bonds formed at the interface of TLR7 and UNC93B1.

| **TLR7 residues** | | | **UNC93B1 residues** | | | **Wild type** | **Mutant** |
| --- | --- | --- | --- | --- | --- | --- | --- |
| Res | Main chain/  sidechain | atom | Res | Main chain/  sidechain | atom |  |  |
| THR788 | Side | Oγ1 | GLU96 | Main | O | 13.53 | 18.12 |
| THR788 | Side | Oγ1 | VAL97 | Main | O | 8.01 | - |
| CYS789 | Main | N | VAL97 | Main | O | 55.33 | 36.4 |
| GLY818 | Main | N | GLU96 | Side | Oε1 | - | 5.77 |
| GLY818 | Main | N | GLU96 | Side | Oε2 | - | 5.67 |
| ALA819 | Main | N | THR93 | Side | Oγ1 | 38.93 | 31.73 |
| ASP829 | Side | Oδ2 | ARG281 | Side | Nε | 12.29 | - |
| ASP829 | Side | Oδ1 | ARG281 | Side | Nε2 | 11.08 | - |
| ASP829 | Side | Oδ1 | ARG281 | Side | Nε | 9.12 | - |
| ASP829 | Side | Oδ2 | ARG281 | Side | Nη2 | 8.37 | - |
| ASP829 | Side | Oδ2 | THR278 | Side | Oγ1 | - | 5.55 |
| TYR831 | Main | O | SER282 | Main | N | 66.47 | 36.66 |
| TYR831 | Main | O | SER282 | Side | Oγ | 7.71 | 10.35 |
| TYR831 | Side | OH | GLY283 | Main | N | - | 5.37 |
| THR832 | Main | O | LYS98 | Side | Nζ | 5 | - |
| THR832 | Side | Oγ1 | PRO280 | Main | O | 13.89 | 17.1 |
| GLU834 | Side | Oε2 | LYS98 | Side | Nζ | 17.97 | 6.51 |
| GLU834 | Side | Oε1 | LYS98 | Side | Nζ | 15.42 | 5.92 |
| **GLU834** | **Side** | **Oε1** | **ARG157** | **Side** | **Nη1** | **8.45** | **14.34** |
| **GLU834** | **Side** | **Oε2** | **ARG157** | **Side** | **Nη2** | **7.48** | **14.16** |
| **GLU834** | **Side** | **Oε1** | **ARG157** | **Side** | **Nη2** | **6.63** | **12.08** |
| **GLU834** | **Side** | **Oε2** | **ARG157** | **Side** | **Nη1** | **6.64** | **12.58** |
| GLU834 | Side | Oε2 | SER282 | Side | Oγ | 18.86 | - |
| GLU834 | Side | Oε1 | SER282 | Side | Oγ | 14.21 | - |
| ASN839 | Side | Nδ2 | TYR154 | Main | O | 23.21 | 11.75 |
| SER846 | Side | Oγ | SER151 | Side | Oγ | 38.43 | 44.63 |
| SER846 | Side | Oγ | SER151 | Side | Oγ | - | 6.12 |
| HSD861 | Side | Nε2 | TRP137 | Side | Nε1 | 5.81 | 6.89 |
| HSD861 | Side | Nε2 | ARG320 | Side | Nη2 | 5.39 | - |
| LEU862 | Main | O | ARG320 | Side | Nη1 | 14.37 | - |
